# Naftopidil reduced the proliferation of lung fibroblasts and bleomycin‐induced lung fibrosis in mice

**DOI:** 10.1111/jcmm.14255

**Published:** 2019-03-15

**Authors:** Hirokazu Urushiyama, Yasuhiro Terasaki, Shinya Nagasaka, Nariaki Kokuho, Youko Endo, Mika Terasaki, Shinobu Kunugi, Kosuke Makita, Hideaki Isago, Keisuke Hosoki, Kunihiko Souma, Takashi Ishii, Hirotaka Matsuzaki, Yoshihisa Hiraishi, Yu Mikami, Satoshi Noguchi, Hiroyuki Tamiya, Akihisa Mitani, Yasuhiro Yamauchi, Akira Shimizu, Takahide Nagase

**Affiliations:** ^1^ Department of Respiratory Medicine, Graduate School of Medicine University of Tokyo Tokyo Japan; ^2^ Department of Analytic Human Pathology Nippon Medical School Tokyo Japan

**Keywords:** antifibrotic action, G1 cell cycle arrest, lung fibrosis, surfactant protein‐D, α‐1 adrenoceptor antagonist

## Abstract

Naftopidil, an α‐1 adrenoceptor antagonist with few adverse effects, is prescribed for prostate hyperplasia. Naftopidil inhibits prostate fibroblast proliferation; however, its effects on lung fibroblasts and fibrosis remain largely unknown. Two normal and one idiopathic pulmonary fibrosis human lung fibroblast lines were cultured with various naftopidil concentrations with or without phenoxybenzamine, an irreversible α‐1 adrenoceptor inhibitor. We examined the incorporation of 5‐bromo‐2ʹ‐deoxyuridine into DNA and lactic acid dehydrogenase release by enzyme‐linked immunosorbent assay, cell cycle analysis by flow cytometry, scratch wound‐healing assay, and mRNA expressions of type IV collagen and α‐smooth muscle actin by polymerase chain reaction. Effects of naftopidil on bleomycin‐induced lung fibrosis in mice were evaluated using histology, micro‐computed tomography, and surfactant protein‐D levels in serum. Naftopidil, dose‐dependently but independently of phenoxybenzamine, inhibited 5‐bromo‐2ʹ‐deoxyuridine incorporation in lung fibroblasts. Naftopidil induced G1 cell cycle arrest, but lactic acid dehydrogenase release and migration ability of lung fibroblasts were unaffected. Naftopidil decreased mRNA expressions of type IV collagen and α‐smooth muscle actin in one normal lung fibroblast line. Histological and micro‐computed tomography examination revealed that naftopidil attenuated lung fibrosis and decreased serum surfactant protein‐D levels in bleomycin‐induced lung fibrosis in mice. In conclusion, naftopidil may have therapeutic effects on lung fibrosis.

## 1 INTRODUCTION

Idiopathic pulmonary fibrosis is a type of chronic, progressive, and irreversible lung fibrosis characterized by the proliferation of fibroblasts and deposition of extracellular matrix, resulting in the destruction of normal lung architecture and loss of pulmonary function.^1^, ^2^ Pirfenidone^3^ and nintedanib^4^ exhibit inhibitory effects on lung fibrosis; however, continuous administration is often difficult due to the various side effects of these drugs. The most frequent adverse effects are nausea (36%) for pirfenidone^3^ and diarrhea (63%) for nintedanib.^4^ These gastrointestinal events exacerbate the poor nutritional status of patients with idiopathic pulmonary fibrosis. Thus, alternative therapy for lung fibrosis with a lower rate of side effects is needed.

α‐1 adrenoceptor antagonists are generally prescribed for prostate hyperplasia with lower urinary tract symptoms. Naftopidil,^5^ a selective α‐1 adrenoceptor antagonist, is frequently used in Japan due to its few side effects. Naftopidil induces G1 cell cycle arrest in prostate fibroblasts and prostate cancer cells, although this effect was independent of the capacity to antagonize α‐1 adrenoceptors in vitro.^6^ Moreover, naftopidil has been reported to decrease prostate volume in patients with prostatic hyperplasia, which is caused by hyperplasia of stroma cells, including fibroblasts and epithelial cells.^7^ However, little is known about the effects of naftopidil on lung fibroblasts and lung fibrosis. The aim of this study was to examine the efficacy of naftopidil on lung fibroblasts and lung fibrosis.

## 2 MATERIALS AND METHODS

### 2.1 Cell culture

Well‐characterized, cultured human lung fibroblast lines, WI‐38^8^ and TIG‐1‐20,^9^ were obtained from the Health Science Research Resources Bank (Osaka, Japan). The human lung fibroblast line LL97A derived from idiopathic pulmonary fibrosis was acquired from the American Type Culture Collection (Manassas, VA). The cells were cultured in Dulbecco's modified Eagle's medium (DMEM; Wako Pure Chemical Industries, Osaka, Japan) supplemented with 10% foetal bovine serum (FBS), 50 µg/mL streptomycin and 50 U/mL penicillin at 37°C under humidified 5% CO_2_ conditions. Naftopidil was kindly provided by Asahi Kasei Pharma (Tokyo, Japan) and phenoxybenzamine was obtained from Sigma‐Aldrich (St. Louis, MO).

### 2.2 Cell proliferation assays

To investigate the effect of naftopidil on the proliferation of human lung fibroblasts, human lung fibroblast lines were cultured with various concentrations of naftopidil (0‐80 µmol/L in 0.1% dimethyl sulfoxide [DMSO]) for 2 days after passaging. The cells were then fixed and stained with Diff‐Quik (Sysmex, Hyogo, Japan), and counted per 0.16 mm^2^ in three random fields with an optical microscope.

To quantify the effect of naftopidil on cell proliferation, we measured the incorporation of 5‐bromo‐2ʹ‐deoxyuridine (BrdU) into DNA after 24 hours of culture with various concentrations of naftopidil (0‐100 µmol/L in 0.1% DMSO) using enzyme‐linked immunosorbent assays according to manufacturer's instructions (Roche, Basel, Switzerland).

To determine whether the inhibitory effect of naftopidil on cell proliferation was independent of α‐1 adrenoceptor inhibition, we measured the incorporation of BrdU into DNA after 24 hours of culture with 40 µmol/L naftopidil in 0.1% DMSO with or without 4 hours pre‐treatment of phenoxybenzamine (1 µmol/L)—an irreversible α‐1 adrenoceptor inhibitor. We also measured the incorporation of BrdU in the cells treated with 1 µmol/L phenoxybenzamine alone.

### 2.3 Cytotoxic assay

To examine the cytotoxic effect of naftopidil on human lung fibroblasts, we measured lactic acid dehydrogenase (LDH) release from the cells after 6 hours of culture with 40 and 80 µmol/L naftopidil, or DMSO alone, or 2% octylphenol ethoxylate (a non‐ionic surfactant) as a positive control. We used LDH Cytotoxicity Detection Kit according to manufacturer's instructions (Takara Bio Inc, Shiga, Japan).

### 2.4 Cell cycle analysis

Cell cycle analysis was performed by flow cytometry. Human lung fibroblasts were treated with or without 40 µmol/L naftopidil in 0.1% DMSO for 24 hours. The cells were then trypsinized, washed, fixed and stained with propidium iodide according to manufacturer's instructions (BD Biosciences, San Jose, CA). The DNA content of 1 × 10^5^ stained cells was analysed using the BD FACSCanto II flow cytometer (BD Biosciences). The proportions of cells in G0/G1 and S/G2/M phases were calculated using FlowJo software (BD Biosciences).

### 2.5 Scratch wound‐healing assay

Human lung fibroblast lines were grown to subconfluence on 12‐well plates, and then scratch wounded with a yellow pipette tip. Cultured medium was then replaced with DMEM supplemented with 10% FBS with 40 µmol/L naftopidil or DMSO alone. Wound regions were photographed with a microscope after 0 and 12 hours of culture.

### 2.6 Real‐time quantitative reverse transcription–polymerase chain reaction (RT‐QPCR) amplification

To examine the effect of naftopidil on transforming growth factor (TGF)‐β1 stimulation, we measured mRNA expression of α1 chain of type IV collagen and α‐smooth muscle actin (α‐SMA) in human lung fibroblast lines. The cells were cultured for 24 hours with 40 µmol/L naftopidil, or DMSO alone, in the absence or presence of 5 ng TGF‐β1. Total RNA was purified using the RNeasy Mini Kit (Qiagen Sciences, Germantown, MD) according to the manufacturer's instructions. Reverse transcription was performed at 25°C for 10 min, 37°C for 120 min, and 85°C for 4 min using High‐Capacity cDNA Reverse Transcription Kit (Thermo Fisher Scientific, Waltham, MA). After reverse transcription, our RT‐qPCR methods were previously described in detail.^10^ Ready‐to‐use primer and probe sets (Thermo Fisher Scientific): numbers Hs00266237_m1, Hs00426835_g1 and Hs02786624_g1 were used to detect α1 chain of type IV collagen, α‐SMA and glyceraldehyde‐3‐phosphate dehydrogenase (GAPDH). Ratios of mRNA expressions of α1 chain of type IV collagen and α‐SMA to those of GAPDH in the same sample were determined, relative to the ratios for cells cultured with DMSO alone in the absence of TGF‐β1 in the same experiments.

### 2.7 Animal procedures

Wild‐type C57BL/6J mice were purchased from Sankyo Labo Service (Tokyo, Japan). Six‐week‐old mice were subcutaneously implanted with an osmotic pump releasing bleomycin at a continuous infusion rate of 50 µg/hour for 14 days (ALZET, Cupertino, CA, USA). The mice were administrated naftopidil orally (30 mg kg^−1^ d^−1^) as a solute in 0.1 mol/L citrate buffer (pH 4) 5 days a week starting from 3 days before to 28 days after implanting the osmotic pump, after which they were killed under isoflurane anaesthesia. Blood collection was also performed under isoflurane anaesthesia before and 28 days after implanting the osmotic pump. Blood samples were centrifuged to collect serum. All procedures were approved by the Committee on the Ethics of Animal Experiments, Nippon Medical School (approval number: 27‐293, 2016).

### 2.8 Micro‐computed tomography (CT)

Mice micro‐CT was performed with an LCT‐100 micro‐CT System (Aloka, Tokyo, Japan). X‐ray intensity in air was −1,000 Hounsfield units and 0 Hounsfield units in water. CT images were acquired with the X‐ray source biased at 50 kVp and 1 mA. Slice thickness was 0.3 mm. Image size was 480 × 480 and the field of view was 48 mm at a resolution of 0.10 mm per pixel. Chest CT scans were performed on killed mice. Mean CT numbers of the maximum cross section in mice lung were calculated with LCT‐100 micro‐CT System software.

### 2.9 Histological examination

For histological analysis, lungs removed from killed mice were inflated with 4% paraformaldehyde, immersed and fixed with 4% paraformaldehyde and then imbedded in paraffin. Lung sections were stained with haematoxylin and eosin (HE) and elastica Masson‐Goldner (EMG) for the evaluation of collagen deposition. Our immunohistochemistry methods were previously described in detail.^10^ Briefly, to unmask antigenic epitopes, sections for type I collagen staining were treated with 1% pepsin in 0.01 N HCl for 60 min, and sections for α‐SMA staining were heated at 120°C for 20 min with 0.01 mol/L citrate buffer (pH 6.0). Primary antibodies were goat polyclonal anti‐type I collagen (1:300 dilution; SouthernBiotech Associates, Birmingham, AL), and mouse monoclonal anti‐α‐SMA (1:300; Dako, Glostrup, Denmark). Sections were then incubated with Histofine Simple Stain Kits (Nichirei Biosciences, Tokyo, Japan) as second antibodies with peroxidase for 30 min. Peroxidase activity was detected with a solution of 3,3′‐diaminobenzidine and H_2_O_2_, with the counterstain being Mayer's haematoxylin.

### 2.10 Analysis of surfactant protein‐D (SP‐D)

The concentration of SP‐D in mice serum was measured by enzyme‐linked immunosorbent assays. We used the Rat/Mouse SP‐D ELISA Kit (Yamasa Corporation, Chiba, Japan) according to manufacturer's instructions.

### 2.11 Statistical analysis

For each data set, arithmetic means and standard error of the mean (SEM) were calculated and then Student's *t* test was used to compare independent variables. Mann‐Whitney *U*‐test was used to compare mean CT numbers. Statistical analysis was performed with GraphPad Prism 6 (GraphPad Software, La Jolla, CA). *P* values <0.05 were considered statistically significant.

## 3 RESULTS

### 3.1 Effect of naftopidil on cell proliferation in human lung fibroblast lines

**Figure 1:**
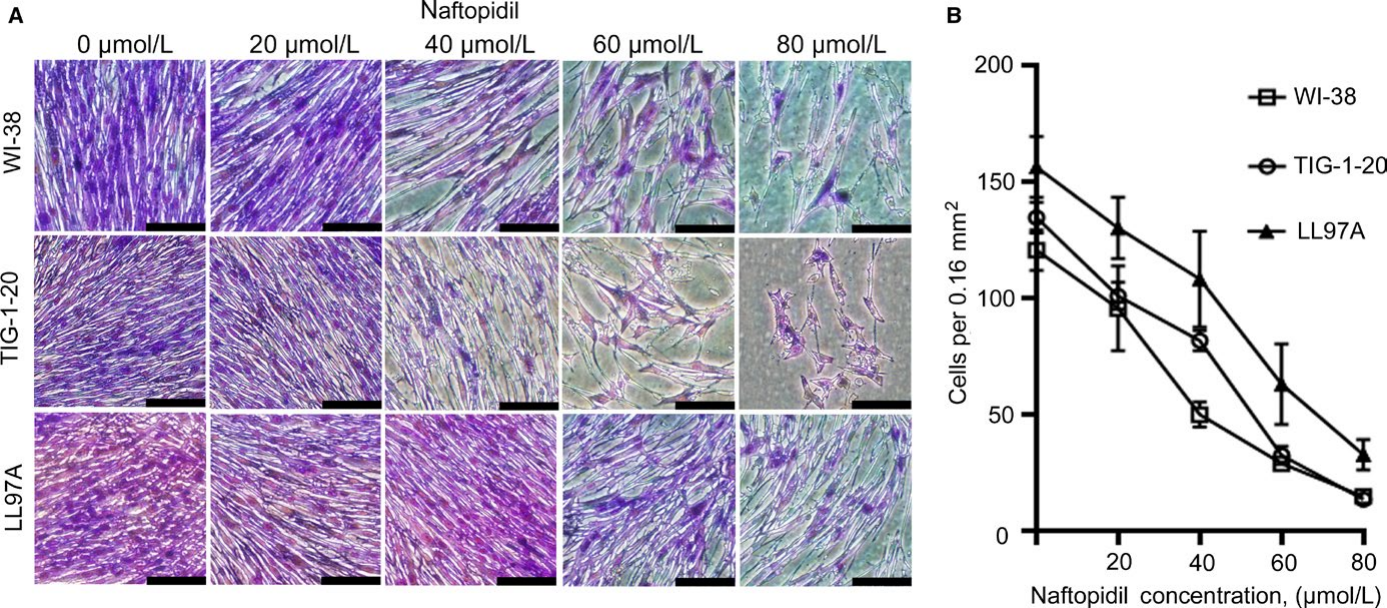
Effect of naftopidil on the proliferation of human lung fibroblast lines. Proliferation of normal lung fibroblast lines (WI‐38 and TIG‐1‐20) and lung fibroblasts derived from idiopathic pulmonary fibrosis (LL97A) in the presence of various concentrations of naftopidil for 48 hours. A, Representative images of cells treated with naftopidil at concentrations ranging from 0 to 80 µmol/L. Scale bars = 200 µm. B, Mean numbers of cells treated with naftopidil at concentrations ranging from 0 to 80 µmol/L per 0.16 mm^2^ in three random fields. Data are means ± SEM from four experiments

**Figure 2:**
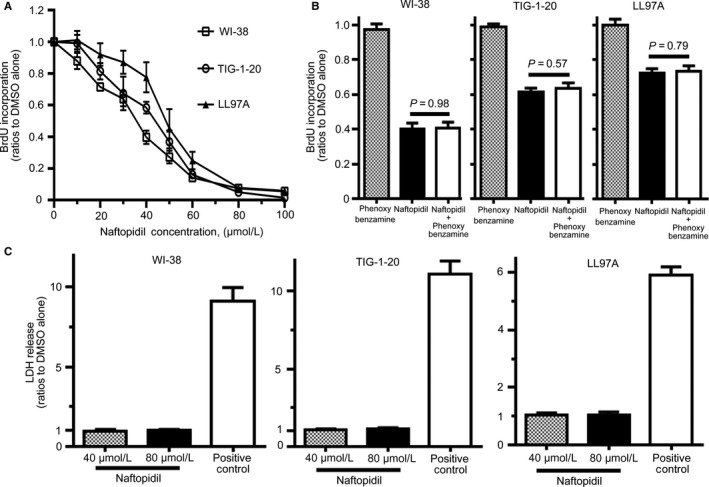
Effect of naftopidil and phenoxybenzamine on the incorporation of 5‐bromo‐2ʹ‐deoxyuridine into the DNA of human lung fibroblast lines. A, Effect of naftopidil on the incorporation of 5‐bromo‐2ʹ‐deoxyuridine (BrdU) in human lung fibroblast lines. BrdU incorporation into DNA in the presence of various concentrations of naftopidil after 24 h was assessed relative to cells treated with dimethyl sulfoxide (DMSO) alone (=1.0) in the same experiments. B, Effect of phenoxybenzamine on BrdU incorporation in human lung fibroblast lines treated with naftopidil. BrdU incorporation into the DNA of cells treated with 1 µmol/L phenoxybenzamine alone or 40 µmol/L naftopidil with or without 4 hours pre‐treatment of 1 µmol/L phenoxybenzamine after 24 hours was assessed relative to cells treated with DMSO alone (= 1.0) in the same experiments. There was no difference between phenoxybenzamine alone and DMSO alone (*P* = 0.91 in WI‐38 cells, *P* = 0.95 in TIG‐1‐20 cells, *P* = 0.97 in LL 97A cells). C, Results of lactate dehydrogenase (LDH) release from human lung fibroblast lines treated with naftopidil. LDH release from the cells treated with 40 or 80 µmol/L naftopidil after 6 hours was assessed relative to cells treated with DMSO alone (=1.0) in the same experiments. Data represent the means ± SEM of four experiments

To investigate the effect of naftopidil on the proliferation of human lung fibroblasts, we examined the proliferation of three human lung fibroblast lines—two normal human lung fibroblast lines (WI‐38 and TIG‐1‐20) and a human lung fibroblast line derived from idiopathic pulmonary fibrosis (LL97A)—in various concentrations of naftopidil. We found that naftopidil decreased the numbers of these three fibroblast lines for 48 hours of culture in a dose‐dependent manner (Figure 1). To quantify the effect of naftopidil on cell proliferation, we measured the incorporation of BrdU into DNA after 24 hours of culture with various concentrations of naftopidil. We found that naftopidil inhibited the incorporation of BrdU in a dose‐dependent manner (Figure 2A).

An earlier study reported that the inhibitory effect of naftopidil on cell proliferation was independent of the capacity to antagonize α‐1 adrenoceptors^6^; to determine this, we examined the ability of phenoxybenzamine, an irreversible α‐1 adrenoceptor inhibitor, to interfere with the inhibitory effect of naftopidil on cell proliferation. Phenoxybenzamine alone did not affect the incorporation of BrdU into the DNA of the cells (Figure 2B). There was no difference in the incorporation of BrdU into the DNA of cells treated with naftopidil with or without pre‐treatment of phenoxybenzamine (Figure 2B).

To examine the cytotoxic effect of naftopidil on human lung fibroblasts, we measured the amount of LDH release from these cells. There was no difference in the amount of LDH release from the cells treated with 40 or 80 µmol/L naftopidil compared with the cells treated with DMSO alone (Figure 2C). Taken together, our findings indicate that naftopidil inhibited the proliferation of human lung fibroblast lines independently of the capacity to antagonize α‐1 adrenoceptors.

### 3.2 Effect of naftopidil on the cell cycle of human lung fibroblast lines

**Figure 3:**
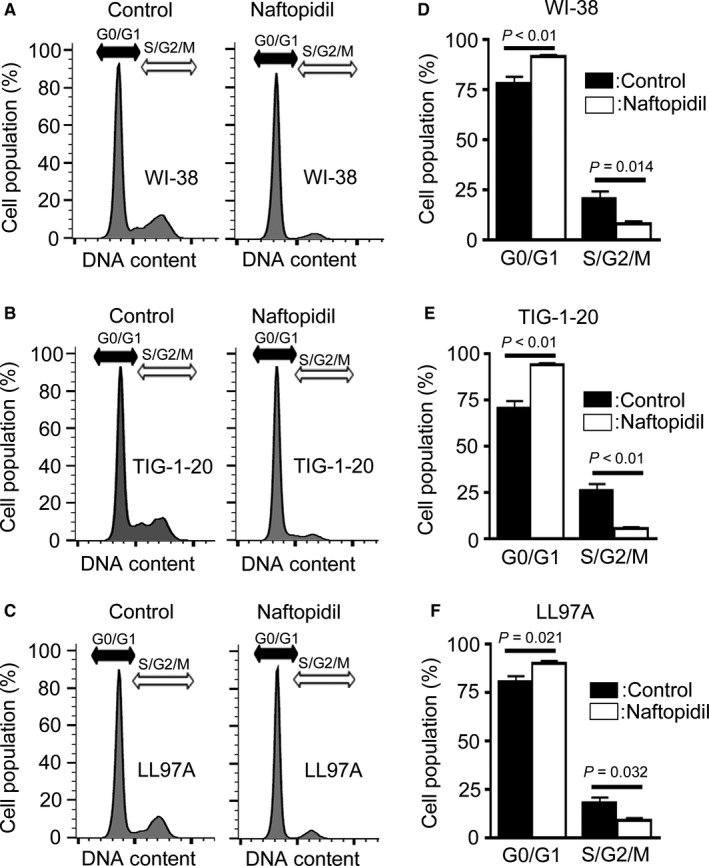
Effect of naftopidil on the cell cycle of human lung fibroblast lines. A‐C, Representative images of cell cycle analysis by flow cytometry of the human lung fibroblast lines (A) WI‐38, (B) TIG‐1‐20, and (C) LL97A treated with naftopidil or without (as control) for 24 h. D‐F, Each column represents the percentage of cells (mean ± SEM of four experiments) in the G0/G1 and S/G2/M phases of the cell cycle in (D) WI‐38, (E) TIG‐1‐20 and (F) LL97A cell lines treated with naftopidil or without (as control)

Previous studies have reported that naftopidil inhibits the growth of human prostate fibroblasts,^6^ human prostate cancer cells,^10^ human renal cell carcinoma cells, and human umbilical vein endothelial cells^11^ by inducing G1 cell cycle arrest. To determine the mechanism by which naftopidil inhibits the proliferation of human lung fibroblasts, we performed flow cytometric analysis of propidium iodide‐stained cells. Compared with control, naftopidil significantly increased the population of human lung fibroblasts in G0 and G1 phase, which decreased in S, G2, and M phase (Figure 3).

### 3.3 Effect of naftopidil on the migration of human lung fibroblast lines

**Figure 4:**
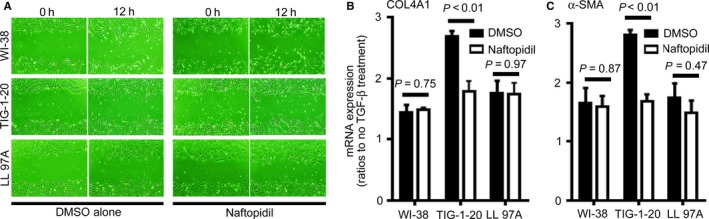
Effect of naftopidil on the migration and responsiveness to TGF‐β stimulation in human lung fibroblast lines. A, Representative images of scratch wound‐healing assay at 0 and 12 h. The left two columns show cells treated with dimethyl sulfoxide (DMSO) alone. The right two columns show cells treated with 40 µmol/L naftopidil. The top, middle and bottom rows displayed WI‐38, TIG‐1‐20 and LL97A cells, respectively. B‐C, Ratios of mRNA expressions of α1 chain of type IV collagen (B, COL4A1) and α‐smooth muscle actin (C, α‐SMA) to GAPDH in the same sample were analysed via real‐time quantitative reverse transcription‐polymerase chain reaction and expressed relative to results for cells cultured without TGF‐β1 (=1.0) in the same experiments. Black columns represented ratios of mRNA expressions in the cells treated with 5 ng/mL TGF‐β and DMSO. White columns represented ratios of mRNA expressions in the cells treated with 5 ng/mL TGF‐β and 40 µmol/L naftopidil. Data are means ± SEM from four experiments

The migration of fibroblasts was thought to play an important role in fibrosis.^12^ We thus examined the effect of naftopidil on the migration of human lung fibroblast lines using a scratch wound‐healing assay. There was no difference in scratch wound‐healing in the presence or absence of naftopidil (Figure 4A).

### 3.4 Effect of naftopidil on the responsiveness to TGF‐β stimulation in human lung fibroblast lines

We previously reported that a lot of myofibroblasts and strong deposition of α1 chain of type IV collagen existed in early fibrotic lesions of idiopathic lung fibrosis, and TGF‐β1 stimulation increased α‐SMA expression and the production of α1 chain of type IV collagen in human lung fibroblasts.^10^ We therefore examined the effect of naftopidil on the responsiveness to TGF‐β1 stimulation in human lung fibroblast lines, by measuring mRNA expressions of α1 chain of type IV collagen and α‐SMA with RT‐qPCR. We found that naftopidil decreased mRNA expressions of α1 chain of type IV collagen and α‐SMA in TIG‐1‐20 cell lines, but not in WI‐38 and LL 97A cell lines (Figure 4B).

### 3.5 Effect of naftopidil on bleomycin‐induced lung fibrosis in mice

We found that naftopidil inhibited the proliferation of three human lung fibroblast lines by inducing G1 cell cycle arrest in vitro. We thus examined the effect of naftopidil on bleomycin‐induced lung fibrosis in mice in vivo. In previous studies, oral naftopidil dosage for mice was reported to be 10 mg to 100 mg kg^−1^ d^−1^.^6^, ^11^, ^13^ In our study, naftopidil was administrated to mice orally (30 mg kg^−1^ d^−1^) as a solute in 0.1 mol/L citrate buffer (pH 4) 5 days a week starting from 3 days before and 28 days after implanting a subcutaneous pump for bleomycin administration.

**Figure 5:**
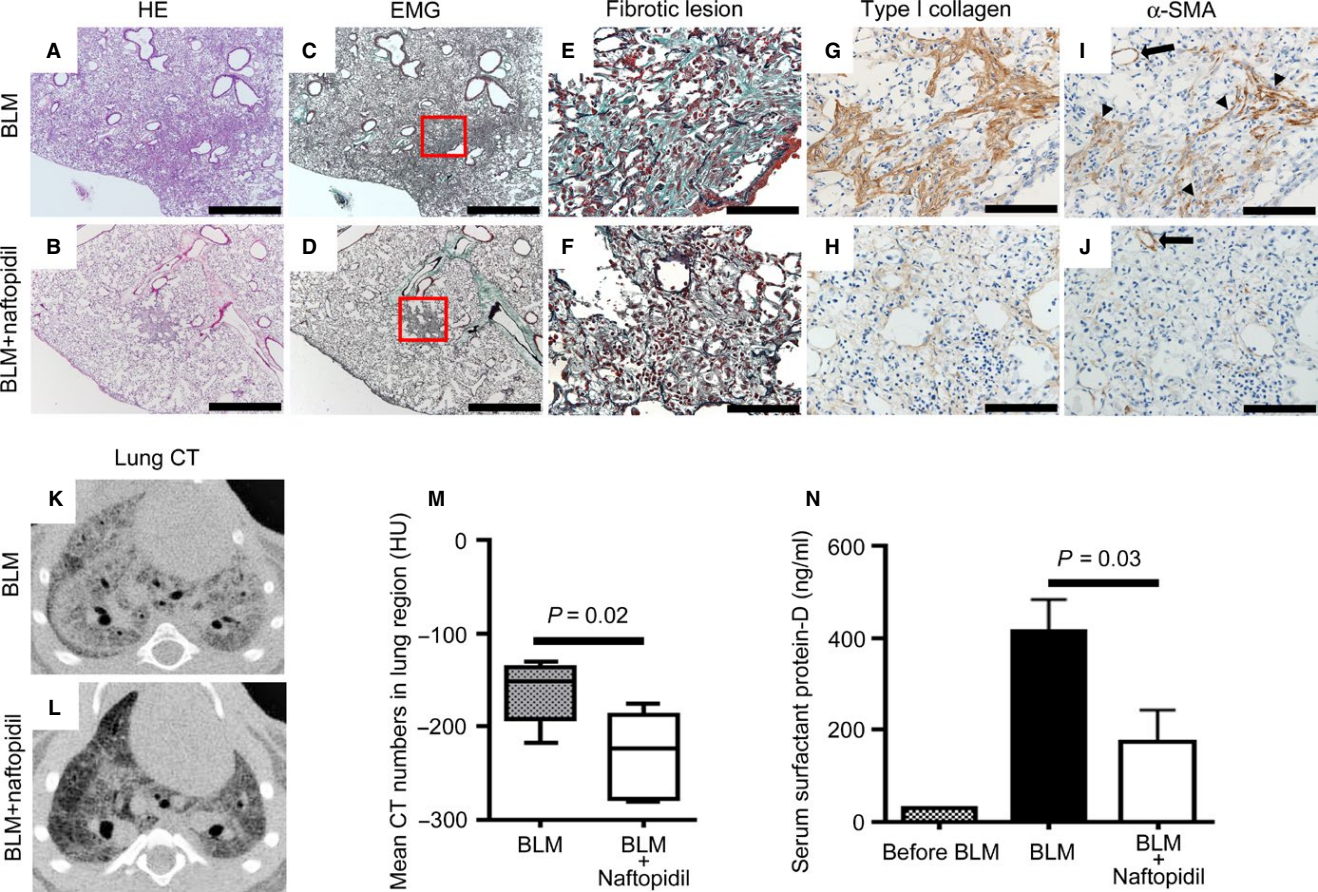
Naftopidil attenuation of bleomycin‐induced lung fibrosis in mice. A‐D, Representative images of haematoxylin and eosin (HE) stained (A, B) and elastica Masson‐Goldner (EMG) stained (C, D) specimens of the mouse bleomycin‐induced lung fibrosis model treated (A, C) without or (B, D) with naftopidil. Scale bars = 1000 µm. E and F, High‐magnification views correspond to the red squares in (C) and (D) of fibrotic lesions of bleomycin‐induced lung fibrosis in mice treated (E) without or (F) with naftopidil. Dark green fibres indicate deposited collagen. Black fibres were elastic fibres. G and H, A lot of type I collagen was observed in lung fibrotic lesions in mice treated without naftopidil (G), while a little type I collagen was observed in mice treated with naftopidil (H). I and J, A lot of α‐SMA positive myofibroblasts were observed in lung fibrotic lesions in mice treated without naftopidil (I), but not in mice treated with naftopidil (J). Arrowheads indicate α‐SMA positive myofibroblasts. Arrows indicate vascular smooth muscle cells as a positive control. Scale bars  = 100 µm. K and L, Representative images of micro‐computed tomography (CT) axial sections of mice lungs 28 d after bleomycin (BLM) administration (K) without or (L) with naftopidil treatment. M, Mean CT numbers of lung region in mice 28 d after bleomycin treatment without (BLM, n = 7) or with naftopidil (BLM + naftopidil, n = 8). N, Serum surfactant protein‐D levels in mice were measured by enzyme‐linked immunosorbent assays before bleomycin administration (before BLM, n = 9), 28 d after bleomycin administration (BLM, n = 7) and bleomycin administration with naftopidil treatment (BLM + naftopidil, n = 8). Data represent the means ± SEM

By means of histological analysis, we confirmed that mice administered bleomycin had marked fibrotic lesions in alveolar areas (Figure 5A,C), while mice treated with bleomycin and naftopidil showed less fibrotic lesions (Figure 5B,D). We also found that more collagen was deposited in fibrotic lesions in bleomycin‐induced lung fibrosis compared with that of the naftopidil treatment (Figure 5E,F). Immunohistochemical analysis revealed that a lot of type I collagen and α‐SMA positive myofibroblasts existed in bleomycin‐induced lung fibrosis (Figure 5G,I), whereas few type I collagen and α‐SMA positive myofibroblasts existed in that of the naftopidil treatment (Figure 5H,J). Lung micro‐CT also demonstrated that mice administered bleomycin were affected with lung fibrosis, whereas mice treated with bleomycin and naftopidil exhibited attenuated lung fibrosis (Figure 5K‐M).

The concentration of SP‐D from patients with idiopathic pulmonary fibrosis and interstitial pneumonia with collagen disease are significantly higher than those of healthy controls.^14^ Serum SP‐D levels have been used as a diagnostic marker for lung fibrosis.^15^ To quantitatively evaluate the extent of fibrosis in the bleomycin‐induced lung, we measured the concentration of mouse serum SP‐D by enzyme‐linked immunosorbent assays 28 days after implanting the subcutaneous pump. We found that bleomycin administration elevated serum SP‐D levels, whereas concurrent naftopidil treatment significantly reduced serum SP‐D levels (Figure 5N). Our findings indicate that treatment with naftopidil reduced the extent of lung fibrosis and SP‐D production in a mouse model of bleomycin‐induced lung fibrosis.

## 4 DISCUSSION

In this study, we demonstrated for the first time that naftopidil inhibited the proliferation of human lung fibroblasts by inducing G1 cell cycle arrest, independently of the capacity to antagonize α‐1 adrenoceptors in vitro. Moreover, we found that naftopidil attenuated bleomycin‐induced lung fibrosis in mice.

Previous studies have reported that naftopidil inhibits the proliferation of various human cells, such as prostate fibroblasts,^6^ human prostate cancer cells,^11^ human renal cell carcinoma cells, human umbilical vein endothelial cells,^13^ and malignant mesothelioma cells.^16^ Focusing on the inhibitory effect of naftopidil on cell proliferation, naftopidil analogues were developed as potential anticancer drugs. HUHS 1015, a naftopidil analogue, efficiently reduced the viability of various human cancer cells, such as those of malignant mesothelioma and lung, gastric, bladder, prostate, and renal cancers.^17^, ^18^ In addition to the role of naftopidil as an anticancer drug, our results suggest that naftopidil is a potential therapeutic drug for lung fibrosis.

Naftopidil is an α‐1 adrenoceptor antagonist with high selectivity for the α‐1A and α‐1D subtypes and an especially high affinity to the α‐1D subtype.^5^ α‐1 adrenoceptors are divided into α‐1A, α‐1B and α‐1D subtypes.^19^ In the prostate gland, α‐1D adrenoceptors are the second most abundantly expressed subtype after α‐1A.^20^ α‐1 adrenoceptor antagonists relax the prostate smooth muscles and decrease urethral resistance, resulting in lower urinary tract symptom relief.^21^ In Japan, naftopidil is widely prescribed for patients with lower urinary tract symptoms associated with prostatic hyperplasia.^22^ Epidemiologically, naftopidil significantly decreased the incidence of prostate cancer in prostatic hyperplasia patients.^23^ Although the mechanisms behind the inhibitory effect of naftopidil on cell proliferation remain unknown compared with the effect on decreasing urethral resistance, both interstitial pneumonias and prostatic hyperplasia are caused by hyperplasia of fibroblasts and epithelial cells and are often complicated with cancers.^2^, ^7^ Because naftopidil decreased prostate volume in patients with prostatic hyperplasia 7 as well as prostate cancer incidence,^23^ naftopidil may be an effective drug for interstitial pneumonias as well as prostatic hyperplasia.

In our results, naftopidil decreased mRNA expression of α1 chain of type IV collagen and α‐SMA in TIG‐1‐20 cell lines, but not in WI‐38 and LL 97A cell lines. In immunohistochemistry, a lot of myofibroblasts existed in bleomycin‐induced lung fibrosis, whereas few myofibroblasts existed in that of the naftopidil treatment. An earlier study reported that naftopidil decreased phosphorylation of Samd2 induced by TGF‐β in HeLa cell line.^23^ Naftopidil thus might attenuate lung fibrosis in part through decreasing α‐SMA expression and collagen expression in some fibroblasts.

In this study, naftopidil was orally administrated to mice (30 mg kg^−1^ d^−1^) based on earlier in vivo mouse studies with oral naftopidil administration in the range of 10 mg to 100 mg kg^−1^ d^−1^.^6^, ^11^, ^13^ Naftopidil is frequently used in Japan because it has few side effects 22 and the 50% lethal dose of naftopidil is over 5000 mg kg^−1^ d^−1^ orally in rats and dogs.^24^ In addition, a 12‐month oral chronic toxicity study of naftopidil revealed that the toxicological no‐effect dose of naftopidil was 25 mg kg^−1^ d^−1^ in rats 25 and dogs.^26^ However, the daily dose of naftopidil for patients with prostatic hyperplasia ranged from 25 mg to 75 mg/d; thus, it is unclear whether our experimental dose (30 mg kg^−1^ d^−1^) is safe for humans. Moreover, to our best knowledge, how naftopidil works for cell cycle arrest has been unclear. Safety concern of high dose naftopidil thus remained. Further validation studies for high doses of naftopidil and its mechanisms of cell cycle arrest are needed.

In conclusion, naftopidil inhibited cell proliferation in human lung fibroblasts by inducing G1 cell cycle arrest independently of the capacity to antagonize α‐1 adrenoceptors in vitro. Moreover, naftopidil reduced the extent of lung fibrosis and SP‐D production in bleomycin‐induced lung fibrosis in mice. Our study demonstrates the first evidence for the antifibrotic effect of naftopidil in lung fibrosis. In the future, further clinical studies will be needed to confirm the role of naftopidil as a therapeutic agent against interstitial pneumonias.

## CONFLICT OF INTEREST

All authors have declared no conflicts of interest.
